# Adaptability and Stability of Proso Millet Grain Yield: A Multi-Environment Evaluation Using AMMI, GGE, and GYT Biplots

**DOI:** 10.3390/plants14172719

**Published:** 2025-09-01

**Authors:** Jin Zhang, Mengyao Wang, Chengyu Peng, Hong Chen, Xiaoning Cao

**Affiliations:** 1 Center for Agricultural Genetic Resources Research, Shanxi Agricultural University, Taiyuan 030031, China; zhangjin09@163.com (J.Z.); wangmengyao0113@163.com (M.W.); 2 College of Agriculture, Shanxi Agricultural University, Jinzhong 030801, China; 18867254933@163.com; 3 Shanxi Seed Industry Development Center, Taiyuan 030006, China; shxpsb@126.com

**Keywords:** proso millet, genotype × environment interaction, AMMI, ASV, GGE biplot, GYT biplot

## Abstract

A pivotal food crop in arid and semi-arid zones, proso millet boasts remarkable economic value, making the breeding of stable high-yield varieties critical for industrial sustainability. This study employed a randomized complete block design to conduct a two-year multi-environment trial on nine new varieties across six representative spring-sown test regions in China. Analytical tools, including additive main effects and multiplicative interaction (AMMI) biplots, AMMI stability values (ASV), genotype and genotype × environment (GGE) models, and genotype by yield–trait (GYT) biplots were utilized to assess genotype–environment (G × E) interactions and screen superior genotypes. AMMI variance analysis showed extremely significant effects of genotype, environment, and G × E on yield traits (*p* < 0.01). G × E principal component analysis identified JS8, PS3, PS6, and PM4 as dominant genotypes. Based on ASV indices, varietal stability rankings were PS5 > YS13 > JS8 > PS3 > PS6 > PM4 > others. GGE analysis indicated PM4 had the broadest adaptability across tested environments, while JS15 exhibited specific adaptability in Datong. Huairen and Shuozhou were validated as ideal testing environments via an ideal environment plot. GYT biplots further confirmed that YS13, JS15, PS3, and PM4 excelled in comprehensive yield–trait combinations. These findings offer a scientific foundation for ecological adaptability evaluation, breeding material selection, and commercial variety promotion.

## 1. Introduction

Proso millet (*Panicum miliaceum* L.), one of the oldest food crops in China, has long occupied an important position in agricultural history [1]. A plant in the Poaceae family, it is characterized by drought tolerance, barren resistance, a short growth period, and strong ecological adaptability [2,3,4]. Globally, it is distributed in semi-arid regions of Europe, with production advantages that make it a key crop in global dryland agriculture. Its grains, after shelling, are nutritionally rich in starch, protein, vitamins, dietary fiber, and various trace elements, holding important application value in dietary structure adjustment and new health food development [5,6,7]. As a link connecting ecology and health, proso millet plays a significant role in China and globally [8,9]. Promoting new varieties is therefore practically significant for advancing the seed industry, meeting consumer needs, protecting the ecological environment, and addressing food security challenges.

Besides genotype and environment, gene–environment interaction (GEI) plays a crucial role in determining crop genetic adaptability and stability. GEI refers to the different responses and deviations of genotypes from the main genetic effects under varying environmental conditions [10], typically influencing crop economic yield and other quantitative traits of agronomic significance [11]. The primary factors affecting GEI include temperature, rainfall, soil physical–chemical properties, etc., while biological stresses such as fungi, bacteria, and viruses are also important barriers [12]. In practical situations, due to insufficient stability caused by genotype-by-environment interactions (GEIs), many environmental factors mask the genetic potential of plants, leading to reduced commercial performance of varieties. For breeders, their main goal is to develop new commercial varieties adaptable to various environments. Therefore, understanding GEI characteristics, especially geographic location, is vital in plant breeding, as GEI may more effectively control the ability to identify and select superior genotypes [13]. However, GEI complicates the screening of superior genotypes and recommendation of specific agroclimatic genotypes, intensifying the difficulty of breeding widely adapted varieties [14]. This necessitates greater caution when evaluating genotypes and selecting optimal ones based on yield and adaptability to target environments.

In this study, multi-environment trials (METs) were adopted as the basic framework, combined with three key statistical methods to analyze GEI effects and evaluate the adaptability and stability of proso millet genotypes. METs are effective for evaluating genotype performance under different environments to identify superior target genotypes and suitable planting regions [15]. A variety of statistical tools and models have been developed to analyze GEI effects under MET conditions [16]. Among them, the additive main effects and multiplicative interaction (AMMI) model integrates analysis of variance with principal component analysis [17,18], focusing on gene–environment interaction effects by separating sum-of-product terms from the additive model interaction term to improve estimation accuracy. Its derived AMMI stability value (ASV) based on interaction principal components 1 and 2 quantifies genotypic stability [19]. The genotype and genotype × environment (GGE) biplot visually displays G and GE effects through graphical patterns (e.g., “which-won-where” mean performance vs. stability) [20,21], enabling intuitive evaluation of genotype superiority and environment representativeness. These two methods are complementary: their combination enhances understanding of GEI, identification of optimal genotypes, and determination of stable high-yield environments, with successful applications in wheat [22], rice [23], maize [24,25], pea [26,27], sorghum [28], sugarcane [29,30], etc.

When evaluating genotypes for multiple traits, the genotype by yield–trait biplot (GYT) provides valuable information [31]. In this approach, genotypic superiority is judged based on the combined level of yield and other traits, rather than single-trait performance. GYT biplots rank genotypes according to their overall advantages in yield–trait combinations, display their trait profiles (i.e., strengths and weaknesses), and thus enable genotype evaluation and recommendations [32]. The model is calculated using the mean genotypic value of yield as a benchmark: if a target trait requires maximization, it is multiplied by yield; if minimization is needed, it is divided by yield. Therefore, the core key trait of this model is yield, and other traits become important only when combined with high yield [31].

Despite the significant advantages of proso millet, its production and application still face prominent challenges. Proso millet is a typical short-day crop with high sensitivity to photoperiod, which leads to problems such as narrow ecological adaptation zones of proso millet varieties and difficulties in promotion and application [33,34]. Also, the yield potential and quality characteristics of existing varieties have not yet fully met the requirements of large-scale and high-quality modern agriculture. Therefore, breeding new varieties with high yield, good quality, and wide adaptability through genetic improvement has become a core approach to break through application bottlenecks and promote the upgrading of the proso millet seed industry. Promoting excellent new varieties can not only directly improve planting benefits but can also better meet consumers’ demand for diversified food materials, play a role in ecological protection in arid and semi-arid regions, and provide sustainable solutions to global food security challenges.

In view of the nutritional value, economic value, and cultural value of Proso millet, improving its yield has become the primary goal of current scientific research personnel. However, the yield of each genotype is always affected by genes, environment, and GEI [30,35]. The use of the performance of multiple genotypes under different environmental conditions can enhance the yield and stability of genotypes [36]. Therefore, it is very important to study the characteristics of GEI and improve the screening efficiency of high-quality genotypes and environments. Against this background, this study aimed to systematically analyze GEI characteristics of proso millet through two-year multi-environment trials in six representative spring-sowing regions, combined with AMMI, GGE, and GYT models. The specific objectives were to: (1) clarify the influence weights of environment, genotype, and GEI on proso millet yield; (2) evaluate genotypic yield stability and ecological adaptability via integrated multi-model analysis; (3) determine ideal test environments for variety screening; and (4) provide a scientific basis for ecological adaptability evaluation, breeding material selection, and commercial variety promotion.

## 2. Results

### 2.1. Analysis of Variance for Yield Traits and the Impact of Environmental Differences on Phenotypes

Based on the results of the analysis of variance (ANOVA) using the AMMI model, there were significant differences in the yield of the nine proso millet genotypes across the six test environments. The effects of genotype (G), environment (E), and genotype × environment interaction (G × E) all reached extremely significant levels (*p* < 0.01, Table 1). The square sum proportion of environmental effects was the highest (63.38%), indicating that yield variation was mainly driven by environmental differences. This was followed by genotypic effects (19.75%), suggesting that both genetic differences among varieties and specific environmental responses jointly influence yield performance. Together, they accounted for 81.13% of the total variance. A relatively high ratio of genetic to environmental effects will help improve selection efficiency and facilitate the selection and identification of favorable genotypes from unfavorable ones.

Considering the impact of GEI on yield, multiplicative effect analysis was used to identify stable genotypes based on the AMMI model. As shown in Table 1, the first two principal components were both significant (*p* < 0.01), explaining 79.89% and 12.36% of the GEI effects, respectively, for a total of 92.25%. The sum of squared residuals was not significant, indicating that these two principal components can effectively reflect the interaction characteristics between varieties and environments and that the model has high accuracy.

### 2.2. Integrated Multi-Model Analysis of Genotypic Yield Performance, Stability, and Adaptability

#### 2.2.1. Basic Characteristics of Yield Mean and Stability

The average yield of the nine genotypes ranged from 3207 to 3871 kg·ha^−1^. PM4 had the highest yield (3871 kg·ha^−1^) and PS5 had the lowest (3207 kg·ha^−1^, Table 2). The yield order from high to low was PM4 > JS15 > PM3 > PS6 > YS13 > PS3 > PS4 > JS8 > PS5. Based on the AMMI stability value (ASV), the stability ranking was PS5 > YS13 > JS8 > PS3 > PS6 > PM4 > PM3 > JS15 > PS4. The smaller the ASV value, the less the variety is affected by environmental fluctuations. PS5 (27.25) and YS13 (31.62) had the best stability, while PS4 (107.78) had the worst. Genotypes with IPCA scores close to 0 (such as PS5 and YS13) were less affected by environmental fluctuations, which was consistent with the ASV results.

#### 2.2.2. Synergistic Verification of Stability and Adaptability by AMMI and GGE Models

**Figure 1:**
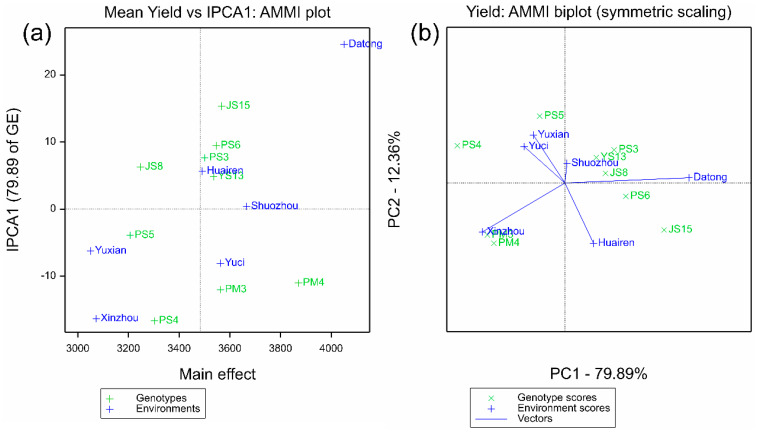
AMMI biplot analysis for nine proso millet genotypes across six test environments. (**a**) AMMII biplot showing the relationship between mean yield (kg ha^−1^, *x*-axis) and interaction principal component analysis 1 (IPCA1, *y*-axis, explaining 79.89% of G × E variation). Symbols: “×” represents genotypes; “+” represents environments. Genotypes with IPCA1 scores close to 0 (e.g., JS8, PS3) exhibit high stability. (**b**) AMMIII biplot based on IPCA1 (*x*-axis, 79.89%) and IPCA2 (*y*-axis, 12.36%). Vector angles between genotypes and environments indicate the strength of G × E interaction (smaller angles = weaker interaction).

**Figure 2:**
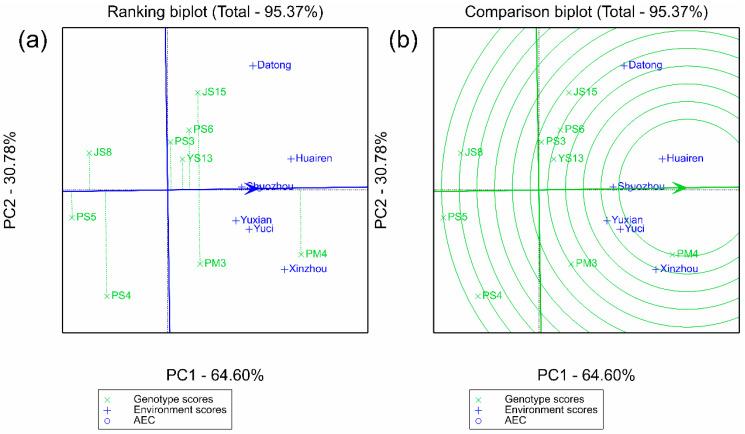
GGE biplots for mean performance vs. stability of nine proso millet genotypes across six test environments. (**a**) Genotype ranking view: the *x*-axis is PC1 (64.60% of total variation), and the *y*-axis is PC2 (7% of total variation). The average environment coordinate (AEC) axis (dashed line) runs from the origin through the average environment, with genotypes closer to the AEC arrow tip showing higher mean yield. Projection length of genotypes on the AEC perpendicular reflects stability (shorter projection = higher stability). (**b**) Ideal genotype view: The “ideal genotype” (center of the circle) is defined by high mean yield and high stability. Genotypes closer to this ideal point (e.g., PM4, PS3) are superior in both yield and stability. Symbols: “×” denotes genotypes; “+” denotes environments.

The AMMII biplot in Figure 1a shows that JS8, PS3, PS6, and PM4 had IPCA1 scores close to 0 (absolute value < 10), and their average yields were higher than the total mean (3536 kg·ha^−1^), showing the characteristic of “high yield and stability.” In contrast, JS15 and PS4 had absolute IPCA1 scores > 15; although their yields were relatively high (JS15 was 3568 kg·ha^−1^). They were significantly affected by the environment and had poor stability. This result was consistent with the “average environment coordinate (AEC)” analysis of the GGE biplot: the stability ranking in GGE (PS5 > YS13 > JS8 > PS3 > PS6 > PM4 > PM3 > JS15 > PS4) was highly consistent with the ASV ranking of AMMI. Moreover, the projections of PM4 and PS3 on the AEC axis were short and located in the high-yield direction, which further verified their “high yield + stability” characteristics (Figure 2a).

**Figure 3:**
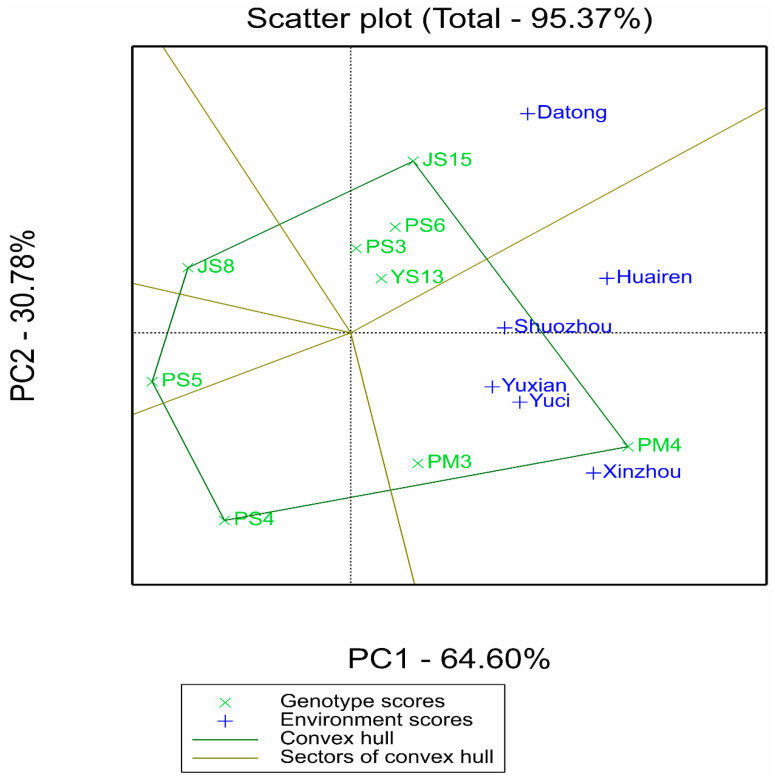
Polygon view of GGE biplot for proso millet yield (kg ha^−1^) across nine genotypes and six environments. The *x*-axis and *y*-axis represent the first two principal components (PC1 and PC2) of the GGE model, explaining 64.60% and 30.78% of total variation, respectively. Polygon vertices indicate genotypes with the highest yield in specific environment sectors: PM4 dominates in Xinzhou, Yuxian, Yuci, and Shuozhou; JS15 dominates in Datong. Symbols: “×” represents genotypes; “+” represents environments.

The GGE “which-won-where” polygon plot in Figure 3 reveals the specific adaptation regions of varieties: PM4 performed optimally in the southern environments with high rainfall and high temperature (Xinzhou, Yuxian, Yuci) and Shuozhou (medium rainfall), while JS15 dominated the advantage sector in the northern Datong pilot with low rainfall and low temperature, indicating that these two varieties are adapted to different ecological regions. In contrast, JS8, PS3, and PS6 performed well in multiple sectors without obvious specificity, reflecting their wide adaptability. Combined with the clustering relationship between varieties and environments in the AMMIII biplot (Figure 1b), the vector angles between JS8, PS3, PS6 and most environments are small. This further supports their wide adaptability characteristics.

#### 2.2.3. Supplementary Analysis of Comprehensive Multi-Trait Advantages by GYT Model

**Figure 4:**
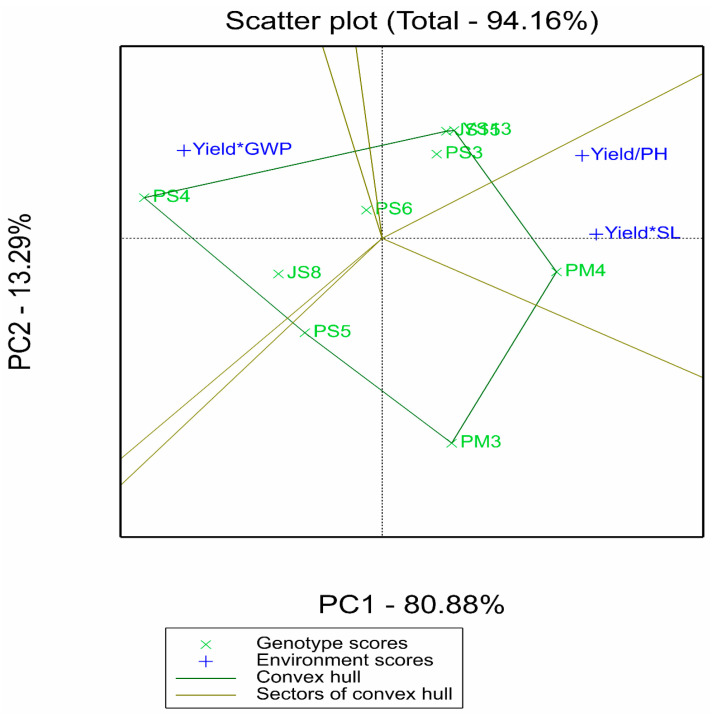
Polygon view of the genotype by yield–trait profile (GYT) biplot.

The GYT biplot in Figure 4 focuses on the combined effects of yield with plant height (PH), spike length (SL), and grain weight per spike (GWP). These traits were selected based on their direct association with yield formation and agronomic significance in proso millet breeding: plant height (PH) is linked to lodging resistance, spike length (SL) correlates with potential grain number per spike, and grain weight per spike (GWP) directly contributes to final yield [5,7]. While other traits such as growth period, thousand-grain weight, and drought tolerance-related indices may also influence adaptability, they were not included due to the focus on core yield–component traits in this study, which is a limitation to be addressed in future research.

**Figure 5:**
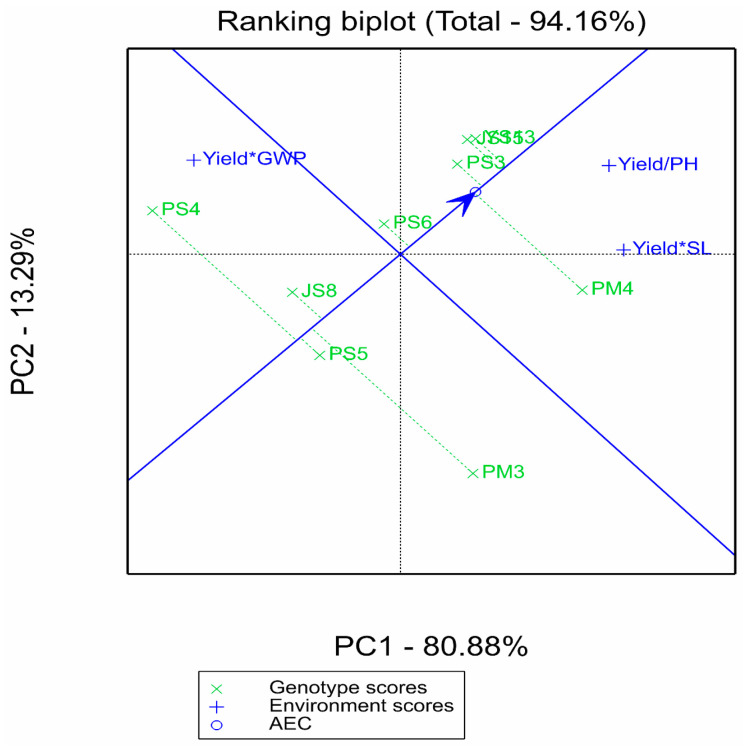
Mean vs. stability view of the genotype by yield–trait profile (GYT) biplot.

The polygon plots intuitively present the comprehensive advantages of genotypes: PM4 performed optimally in the “Yield/SL” (yield–spike length) and “Yield/PH” (yield–plant height) combinations, reflecting spike length advantages and lodging resistance potential on the basis of high yield. Although PS4 stood out in “Yield*GWP” (yield × grain weight per spike), combined with the results of AMMI and GGE, it showed insufficient overall yield and stability, resulting in limited comprehensive value. The mean and stability plot in Figure 5 further reveals that YS13, JS15, PS3, and PM4 were close to the average test axis (ATA), indicating good balance in their yield–trait combinations. This complements the “high-yield and stable” characteristics identified by AMMI/GGE, thereby improving the dimension of genotype evaluation.

### 2.3. Discrimination, Representativeness of Test Environments, and Determination of Ideal Environments

**Figure 6:**
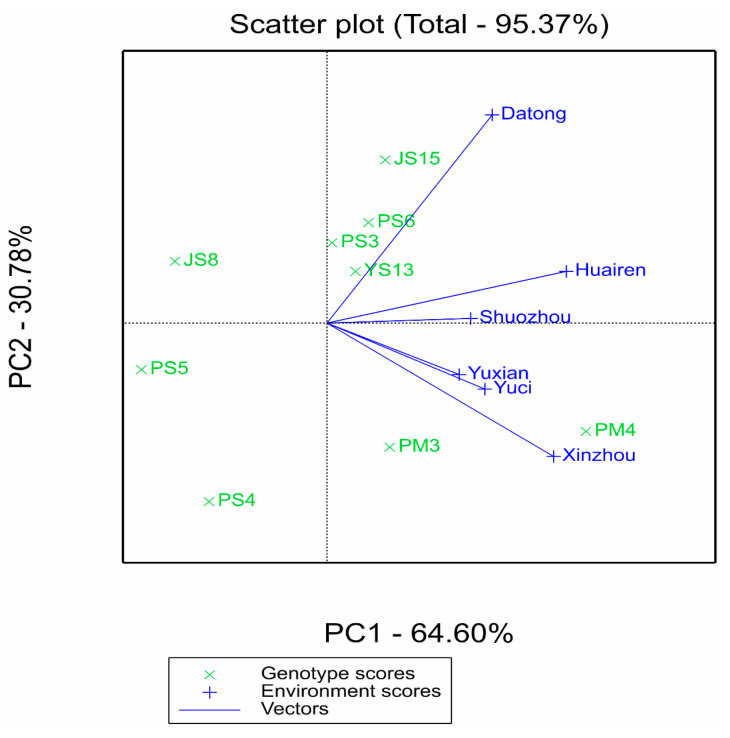
Vector view of the environments for seed yield (kg ha^−1^) of nine genotypes across six test environments.

**Figure 7:**
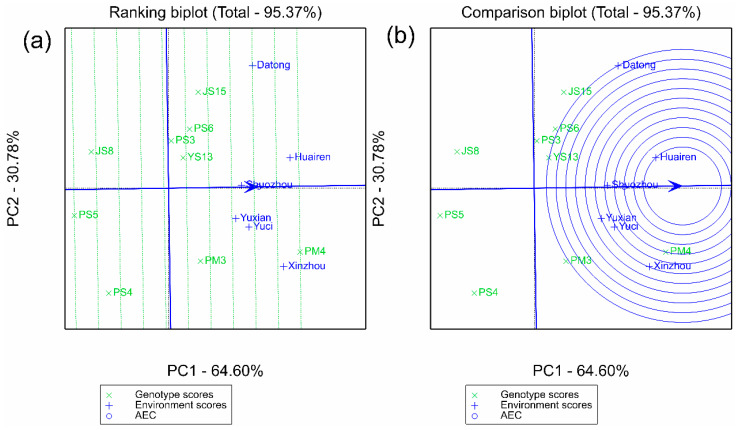
Assessment of the environment based on GGE biplot. (**a**) Discriminativeness and representativeness view of the GGE biplot for 6 test environments; (**b**) ranking environments with ideal environments.

The GGE environment vector plot in Figure 6 shows that the angles between the six environments are all < 90°, indicating a significant positive correlation, which means that the environments have consistent selection directions for varieties, but differ in intensity. In Figure 7a, Datong has the longest vector (the strongest discrimination ability), which can effectively identify genotypic differences; Shuozhou has the smallest angle with the average environment axis (AEA) (the strongest representativeness), which can reflect the overall characteristics of the main spring-sown proso millet producing areas. The ideal environment ranking plot (Figure 7b) integrating discrimination and representativeness shows that Huairen > Shuozhou > Yuci > Yuxian = Xinzhou > Datong. Huairen, a typical area with medium rainfall and medium altitude, has both strong discrimination ability and high representativeness and can be used as the core pilot for subsequent variety screening.

### 2.4. Comprehensive Screening of Superior Genotypes

Integrating the results of AMMI, GGE, and GYT models, the core conclusions were cross-validated through multiple plots: the GGE polygon plot (Figure 2) showed that PM4 performed optimally in the sectors where five pilot sites were located, and JS15 exclusively occupied the Datong sector, corresponding to wide-area adaptation and northern-specific adaptation respectively; Both had high yield and stability in AMMI (ASV < 70), no specific adaptation sectors in GGE (wide adaptability), and significant multi-trait combination advantages in GYT, thus being confirmed as widely adaptable and high-yielding types. JS15, due to its specific adaptation performance in Datong, is suitable for promotion in the northern low-rainfall and low-temperature areas. PS5 had the best stability (ASV = 27.25), but the lowest yield, and could be used as a control for stress-tolerance research.

## 3. Discussion

Breeding proso millet varieties adapted to diverse environments is critical for expanding planting scales, but genotype × environment interaction (GEI)—driven by climate, soil, and biological stresses [27]—complicates the identification of superior genotypes. The grain yield of each genotype is jointly affected by genotype (G), environment (E), and GEI [22,37]. To improve the stability and yield of genotypes, testing multiple varieties under diverse environments is necessary to identify superior genotypes and suitable regions [38,39]. Multi-model integration enhances GEI analysis accuracy, with the additive main effects and multiplicative interaction (AMMI) model and genotype × environment (GGE) biplot being complementary tools [40,41]. AMMI decomposes GEI into orthogonal interaction principal components (IPCA1 and IPCA2), enabling quantification of genotypic stability via IPCA scores (near 0 indicates low environmental sensitivity) [42,43]. GGE biplots visualize “genotype + GEI” effects through polygon plots (specific adaptation) and vector analysis (environmental representativeness), with stability reflected by projection length on the average environment axis (AEA) [20,44,45]. Their integration strengthened our stability evaluations by combining GEI decomposition (AMMI) and visual adaptability mapping (GGE).

Environmental vector length in GGE biplots indicates discriminating power (ability to reveal genotypic differences), while the angle with the AEA reflects representativeness (alignment with average production conditions) [46,47]. Datong’s long vector showed strong discrimination, likely due to its unique low-rainfall, low-temperature conditions amplifying stress-tolerance variations. Shuozhou’s small AEA angle best represented core spring-sown regions, mirroring average rainfall and altitude. Balancing these traits, Huairen and Shuozhou emerged as ideal trial sites for elite genotype screening (Figure 7b) [20].

This GEI pattern aligns with multi-environment trials in related cereals. Environmental factors dominate yield variation, with GEI accounting for 10%–15% of total variance (consistent with our 12.39%) [3,47]. High-stability genotypes (e.g., YS13 here and in foxtail millet trials [47]) share physiological resilience (e.g., stable chlorophyll retention under stress), supporting universal stress-tolerance mechanisms. In our GGE analysis, YS13, PS3, PS6, and PM4 had short AEA projections and aligned with the high-yield direction, confirming broad adaptability [48,49,50].

Their stable performance across rainfall (247–563 mm) and temperature (6.0–10.5 °C) gradients suggests low sensitivity to environmental fluctuations, driven by distinct biological and agronomic traits. PM4 exhibited the highest yield (3871 kg·ha^−1^) and broad adaptability, likely due to its optimal agronomic configuration—moderate plant height (75–80 cm) reduces lodging risk across environments [7], while longer spike length (18–20 cm) enhances sink capacity for a stable grain set [4]—and its consistent performance in both high-rainfall (Xinzhou, Yuxian) and medium-rainfall (Shuozhou) regions aligns with previous findings that balanced source–sink relationships to improve yield stability in proso millet [3]. PS3 maintained stable yield across sites, associated with its high grain weight per spike (2.8–3.2 g) and efficient photosynthetic allocation [4], a trait that ensures stable assimilate supply to grains even under variable moisture conditions, a key adaptive mechanism in cereals [25]. YS13 ranked top in stability (ASV = 31.62) due to strong physiological resilience, as it retains higher chlorophyll content under drought stress (consistent with proso millet drought-tolerance studies [8]) and exhibits efficient osmotic adjustment via proline accumulation, mitigating the impact of environmental fluctuations [9]. JS15 showed specific adaptation to Datong (low rainfall, cool temperatures), likely linked to its drought-tolerant traits such as deeper root distribution (enhancing water uptake in arid conditions) and cold tolerance (higher germination rate at 10–15 °C), traits critical for northern semi-arid regions [34]. In contrast, PM3 and JS15 had longer projections, showing instability likely due to their specific responses to extreme moisture or temperature conditions. Though PS5 and JS8 had short projections, they were oriented opposite to the AEA, indicating high stability, but poor yield, likely trade-offs between stress tolerance and productivity. Since ideal genotypes should combine high yield and stability [15], PM4, YS13, PS6, and PS3 emerged as top candidates, balancing performance across environmental gradients.

Stability rankings differed slightly among ASV (AMMI-based), GGE, and GYT models due to their distinct analytical frameworks and focus on genotype–environment interaction (GEI) components. The AMMI stability value (ASV) quantifies stability by integrating the magnitudes of interaction principal component 1 (IPCA1) and IPCA2, emphasizing the decomposition of GEI into orthogonal components to isolate genotypic stability from environmental noise [19,35], a method that prioritizes minimizing GEI magnitude, but does not directly incorporate mean yield. GGE biplots focus on “genotype + GEI” effects (excluding environmental main effects) and evaluate stability relative to an “ideal genotype” that balances high mean yield and low GEI, with stability reflected by projection distance from the average environment axis (AEA) [20,41], meaning genotypes performing well in representative environments may rank higher here. Genotype × yield × trait (GYT) biplots integrate yield with key agronomic traits (e.g., yield/spike length), defining stability as multi-trait balance rather than GEI magnitude alone [31,51], leading genotypes strong in yield–trait combinations to often show higher stability rankings in GYT. These differences arise from the methods’ distinct focuses—GEI decomposition, genotype–environment alignment, and multi-trait integration—highlighting that stability is a multi-dimensional concept requiring multi-model validation.

In practical breeding, linking stability to specific morphological traits enhances the actionable value of our findings for proso millet improvement. High-yield and stable genotypes like PM4 and PS3 owe their consistent performance to distinct adaptive traits. PM4’s moderate plant height (75–80 cm) minimizes lodging risk across variable rainfall conditions [7], while its longer spike length (18–20 cm) strengthens sink capacity for stable grain set [4]. PS3 maintains stability through high grain weight per spike (2.8–3.2 g) and efficient photosynthetic assimilation allocation, a trait critical for buffering environmental fluctuations in cereals [25]. Breeders should prioritize retaining these traits via marker-assisted selection for linked genetic loci. For low-yield, but stable varieties such as PS5—with the lowest ASV (27.25), but lowest yield (3207 kg·ha^−1^)—targeted improvement is needed: crossbreeding with high-grain-weight genotypes like PS3 can enhance grain weight per spike (currently 2.1 g), while selecting for loci associated with low IPCA scores [19] will preserve its stable genotype–environment interaction response. Optimizing plant height to balance stress tolerance and productivity further improves its commercial potential. These recommendations align with broader patterns in cereal multi-environment trials [3,47], where trait-based selection consistently enhances stability and yield synergy.

The GYT biplot indicates enhanced genotype selection by evaluating multi-trait integration, ranking genotypes based on yield combined with traits like plant height, spike length, and grain weight per spike while visualizing trait correlations [31]. This practical and inclusive approach clarifies each genotype’s strengths and weaknesses [51]. In this study, all yield–trait combinations showed significant correlations (positive or negative) in the GYT biplot, reducing false-positive selections [52]. Such multi-trait evaluation has been validated in other crops [51,52,53], and here YS13, JS15, PS3, and PM4 stood out for balanced yield–trait performance, reinforcing their value in both high-yield scenarios and trait-specific improvement programs.

## 4. Materials and Methods

### 4.1. Test Germplasm Materials and Trial Sites

This experiment was conducted during the proso millet growing season from May to October in 2019 and 2020, with a total of 6 trial sites distributed in Datong, Shuozhou, Huairen, Yuxian, Xinzhou, and Yuci. These sites are representative of spring-sown Proso millet planting areas in China, and their geographic, climatic, soil physical and chemical properties are shown in Table 3.

The soil types across sites were dominated by loam and sandy loam, with pH ranging from 7.2 to 8.1 (slightly alkaline). Soil fertility indicators (0–20 cm layer) showed organic matter content of 12.5–18.3 g·kg^−1^, total nitrogen of 0.82–1.15 g·kg^−1^, available phosphorus (P_2_O_5_) of 15.6–23.8 mg·kg^−1^, and available potassium (K_2_O) of 120–165 mg·kg^−1^.

A total of 9 tested varieties were included, namely Jinshu 8 (JS8), Yanshu 13 (YS13), Jinshu 15 (JS15), Pingshu 3 (PS3), Pingshu 4 (PS4), Pingshu 5 (PS5), Pingshu 6 (PS6), Pinmi 3 (PM3), and Pinmi 4 (PM4). These varieties have rich genetic background diversity, covering different breeding pedigrees such as improved lines of local varieties and hybrid breeding lines. They show significant differences in key agronomic traits such as plant type, growth period, and stress resistance, and can well reflect the characteristics of representative materials in current proso millet breeding.

### 4.2. Experimental Design

The experiment adopted a randomized block design with 3 replications. Each plot had an area of 66.7 m^2^ consisting of 13 rows, and the planting density was 45 plants per hectare. The sowing dates were May 8–12 in 2019 and May 10–15 in 2020, with mechanical drill sowing at a depth of 3–4 cm. Harvesting was conducted from September 25 to October 5 in 2019 and September 28 to October 8 in 2020, depending on maturity. For fertilization management, basal fertilizers were applied during soil preparation, including 30 t/hm^2^ of decomposed organic fertilizer and 300 kg/hm^2^ of nitrogen–phosphorus–potassium compound fertilizer (N:P_2_O_5_:K_2_O = 15:15:15), and 150 kg/hm^2^ of urea was top-dressed at the jointing stage. In terms of irrigation, supplementary drip irrigation was only conducted when severe drought occurred during the growing period (relative soil water content < 50%) with a single irrigation of 45 m^3^/hm^2^, and natural precipitation was relied on at other times. At maturity, plant height, panicle length, and panicle grain weight were investigated. Indoor seed testing was performed following a unified method. During harvest, the edge rows of each plot were excluded from yield calculation, with the actual harvested area being 45 m^2^.

### 4.3. Data Analysis

The data collected from six environments and nine varieties were used to analyze the significance levels of genotype, environment, and G × E interaction effects. AMMI, GGE, and GYT biplot analyses were conducted using GenStat 21st statistical software [54]. Prior to data analysis, the Shapiro–Wilk test was used to assess the normality of yield and agronomic trait data. The results showed that all data conformed to a normal distribution (*p* > 0.05), so no transformation was needed for subsequent statistical analysis.

The AMMI model is as follows:

$$Y_{\mathrm{ij}}\;=\;\mu \;+\;g_{i}\;+\;e_{j}\;+\;\sum \lambda_{k}\alpha_{ik}\delta_{jk}+\varepsilon ij$$

where $Y_{ij}$ is the yield of the i-th genotype in the j-th environment, μ is the grand mean, $g_{i}$ is the deviation of the mean of genotype i from μ, $e_{j}$ is the deviation of the mean of environment j from μ, $\lambda_{k}$ is the eigenvalue of the k-th principal component analysis, $a_{ik}$ and $\delta_{kn}$ are the principal component scores of the i-th genotype and j-th environment for the k-th principal component, respectively, and $\varepsilon_{ij}$ is the residual term.

The ASV calculation formula is as follows:

$$ASV=\sqrt{{\left(\frac{IPCA1\;sum\;of\;square}{IPCA2\;sum\;of\;square}\times IPCA1\;score\right)}^{2}+{IPCA2\;score}^{2}}$$

The GGE double standard graph model is as follows:

$$Y_{ij}=\mu +a_{j}+\lambda 1\rho i1\theta j1+\lambda 2\rho i2\theta j2+\varepsilon ij$$

where $Y_{ij}$ is the yield of the i-th genotype in the j-th environment, μ is the grand mean, $e_{j}$ is the main effect of the j-th environment, λ_1_ and λ_2_ are the singular values of the first two principal components PCA1 and PCA2, ρ_i1_ and ρ_i2_ are the eigenvectors of PCA1 and PCA2 for the i-th genotype, θ_j1_ and θ_j2_ are the eigenvectors of PCA1 and PCA2 for the j-th environment, and εij is the residual that cannot be explained by genotypic (G) or genotype–environment interaction (GE) effects.

Before analyzing the GYT combination data, it was necessary to normalize the data so that the mean of each trait or yield–trait combination became 0 and the variance became unity. This standardization was performed as follows:

$$Y_{mn}=\frac{O_{mn}-O_{n}}{S_{n}}$$

where Y_mn_ is the standardized value of trait or yield–trait combination n for genotype m, O_mn_ is the original value of trait or yield–trait combination n for genotype m, O_n_ is the mean value of trait or yield–trait combination n for genotype m, and S_n_ is the standard deviation of trait or yield–trait combination n.

## 5. Conclusions

In this study, a two-year multi-environment experiment was conducted on nine new proso millet varieties in six representative spring-sowing producing areas in China. Combined with the analysis of AMMI, GGE, and GYT biplots, it was found that the genotype, environment, and genotype × environment interaction effects on yield traits all reached extremely significant levels. Among the total sum of squares of yield differences, GEI accounted for 12.39%, indicating that the specific response between genotype and environment was an important source of yield variation. The sum of squares of environmental effects accounted for 63.38% of the total variation, which was the main factor driving the differences in yield phenotypes, and Huairen and Shuozhou were ideal test environments. Comprehensively, PS3 and PM4 were high-yielding, stable, and widely adaptable varieties. JS15 showed specific adaptability in Datong area, and YS13, JS15, PS3, and PM4 had prominent comprehensive advantages in yield–trait combinations. These results provide a scientific basis for the identification of ecological adaptability of proso millet, the screening of breeding candidate materials, and the promotion of commercial varieties.

## Figures and Tables

**Table 1:** AMMI analysis of variance for proso millet yield (kg·ha^−1^) of nine genotypes in six environments.

Source	d.f.	s.s.	m.s.	v.r.	F pr	%GE	%SS
Total	107	20,389,988	190,561				
Treatments	53	19,477,392	367,498	32.19	<0.001		95.52
Genotypes	8	4,027,798	503,475	44.1	<0.001		19.75
Environments	5	12,923,233	2,584,647	42.54	<0.001		63.38
Block	6	364,561	60,760	5.32	<0.001		1.79
Interactions	40	2,526,361	63,159	5.53	<0.001		12.39
IPCA 1	12	2,018,257	168,188	14.73	<0.001	79.89	
IPCA 2	10	312,356	31,236	2.74	0.0095	12.36	
IPCA 3	8	141,735	17,717	1.55	0.1648	5.61	
Residuals	10	54,013	5401	0.47	0.8992	2.14	
Error	48	548,036	11,417				

Note: d.f. = degrees of freedom; s.s. = sum of squares; m.s. = mean squarde; v.r. = variance ratio; F pr = significance probability; %GE = explanation rate of each principal component in G × E; %SS = percentage of each variation source in the total sum of squares.

**Table 2:** Genotype mean values, IPCA scores, and stability values for yield across all environments.

Genotype	Number	Stability (ASV)	Stability Rank	Yield (kg ha^−1^)	Yield Rank	IPCAg1	IPCAg2	IPCAg3
JS15	1	99.45	8	3568	2	15.35012	−7.27391	−3.11981
JS8	2	40.63	3	3248	8	6.28441	1.50112	−0.47202
PM3	3	77.98	7	3563	3	−12.00416	−8.07218	6.20031
PM4	4	71.81	6	3871	1	−11.01911	−9.35572	1.32932
PS3	5	49.65	4	3501	6	7.64295	5.13867	1.11319
PS4	6	107.78	9	3303	7	−16.65642	5.78785	−11.24808
PS5	7	27.25	1	3207	9	−3.90099	10.36699	7.37735
PS6	8	61.09	5	3547	4	9.44874	−2.05827	−4.66214
YS13	9	31.62	2	3536	5	4.85447	3.96544	3.48188

**Table 3:** The main geographic characteristics of six environments used in this study.

Sites	Altitude (m.a.s.l.)	Longitude	Latitude				2019		2020	
				Soil Type	Soil PH	Organic Matter (g·kg^−1^)	Annual Rainfall (mm)	Mean Temperature (°C)	Annual Rainfall (mm)	Mean Temperature (°C)
Datong	1050	113.349863° E	40.184444° N	Sandy loam	7.8	12.5	256.9	6.5	247	6.5
Huairen	1150	113.154957° E	39.838817° N	Loam	7.2	18.3	365	6	333.5	6.8
Shuozhou	1147	112.457984° E	39.407646° N	Loam	7.5	15.7	371.9	7	423	7
Xinzhou	790	112.712176° E	38.445148° N	Sandy loam	8.1	13.2	529.7	8.5	458.9	8.5
Yuxian	1079	113.423528° E	38.053179° N	Loam	7.6	16.5	563	8.5	547.1	10.5
Yuci	795	112.792022° E	37.712020° N	Sandy loam	7.4	14.8	495	10	472	9.8

Notes: m.a.s.l., meter above sea level. Soil physical and chemical properties were determined from 0–20 cm topsoil samples before sowing in 2019.

## Data Availability

The original contributions presented in this study are included in the article. Further inquiries can be directed to the corresponding author.
